# Thapsigargin blocks electromagnetic field‐elicited intracellular Ca^2+^ increase in HEK 293 cells

**DOI:** 10.14814/phy2.15189

**Published:** 2022-05-04

**Authors:** Federico Bertagna, Rebecca Lewis, S. Ravi P. Silva, Johnjoe McFadden, Kamalan Jeevaratnam

**Affiliations:** ^1^ Leverhulme Quantum Biology Doctoral Training Centre University of Surrey Guildford Surrey UK; ^2^ School of Veterinary Medicine Faculty of Health and Medical Sciences University of Surrey Guildford Surrey UK; ^3^ Advanced Technology Institute University of Surrey Guildford Surrey UK; ^4^ School of Biosciences and Medicine Faculty of Health and Medical Sciences University of Surrey Guildford Surrey UK

**Keywords:** calcium, electromagnetic fields, endoplasmic reticulum, intracellular dynamics

## Abstract

Biological effects of electromagnetic fields (EMFs) have previously been identified for cellular proliferation and changes in expression and conduction of diverse types of ion channels. The major effect elicited by EMFs seems to be directed toward Ca^2+^ homeostasis. This is particularly remarkable since Ca^2+^ acts as a central modulator in various signaling pathways, including, but not limited to, cell differentiation and survival. Despite this, the mechanisms underlying this modulation have yet to be unraveled. Here, we assessed the effect of EMFs on intracellular [Ca^2+^], by exposing HEK 293 cells to both radio‐frequency electromagnetic fields (RF‐EMFs) and static magnetic fields (SMFs). We detected a constant and significant increase in [Ca^2+^] subsequent to exposure to both types of fields. Strikingly, the increase was nulled by administration of 10 μM Thapsigargin, a blocker of sarco/endoplasmic reticulum Ca^2+^‐ATPases (SERCAs), indicating the involvement of the endoplasmic reticulum (ER) in EMF‐related modulation of Ca^2+^ homeostasis.

## 1 INTRODUCTION

The last decade experienced an exponential increase in the daily usage of electronic instruments, such as smartphones, laptops and wireless devices, producing a broad range of electromagnetic fields (EMFs) (IARC Working Group on the Evaluation of Carcinogenic Risks to Humans, World Health Organization, and International Agency for Research on Cancer, 2002; Organization, W.H., 2007). This has led to higher intensities and prolonged exposure to users with respect to the electromagnetic radiation produced by these devices. Along with this, there is increasing interest in the biological impact of this exposure within the scientific community.

Numerous investigations have reported an association between chronic exposure to extremely low frequency electromagnetic fields (ELF‐EMFs), up to 300 Hz, and an increased risk of developing childhood leukemia (London et al., 1991; Savitz et al., 1988). Recently the WHO’s International Agency for Research on Cancer listed radio‐frequency electromagnetic fields (RF‐EMFs), ranging from 10 MHz to 300 GHz, as potentially carcinogenic (Baan et al., 2011). On the other hand, an increasing number of studies reported EMFs beneficial role in the treatment of numerous chronic diseases, such as cancer, mood disorders and many forms of neurodegeneration (Jimenez et al., 2018; Martiny et al., 2010; Wyszkowska et al., 2019), paving the way for the therapeutic use of magnetic‐field based techniques, such as Transcranial Magnetic Stimulation and Pulsed Electromagnetic Field (PEMF) stimulation (Hallett, 2000; Markov, 2007).

The biological effects of EMFs are varied and appear to affect cell physiology on many levels. Both ELF‐EMFs and RF‐EMFs have been shown to modulate cell proliferation (Choi et al., 2020; Grassi et al., 2004). They have been linked to direct changes in genetic and protein expression, such as modulation of ion transport rate in different types of ion channels (Czyz et al., 2004; Grassi et al., 2004; Haghani et al., 2013; He et al., 2013; Lacy‐hulbert et al., 1998). Specifically, excitable tissues like those found in the central nervous system are particularly sensitive to EMFs. Here, RF‐EMFs have been linked to abnormal brain development, activation of the autophagic pathway, decreased number and size of synaptic vesicles and reduced ion channels expression (Kim et al., 2018, 2019; Odaci et al., 2008). ELF‐EMFs on the other hand, have been shown to affect synaptic plasticity, increase glutamate and GABA release, increase ROS levels, and facilitate all forms of endocytosis (Balassa et al., 2013; Duan et al., 2014; Morabito et al., 2010; Sun et al., 2016).

Amongst all these effects however, particular emphasis has been placed on the modulation of Ca^2+^ dynamics. Several studies have identified Ca^2+^ channels as the principal modulators of EMF impacts on cellular dynamics (Buckner et al., 2015; Cui et al., 2014). The involvement of Ca^2+^ is remarkable, since this cation plays a primary role as a second messenger in the modulation of a variety of physiological functions, including, but not limited to, gene expression, cell motility and survival, muscle contraction, membrane excitability, neurotransmitter release and stress response (Ebashi & Endo, 1968; Hardingham et al., 1997; Neher & Sakaba, 2008; Nicotera & Orrenius, 1998; Reddy et al., 2011; Tian et al., 2010; Tsien, 1983). It is well established that the relative concentrations of Ca^2+^ in the various subcellular compartments are finely regulated by a set of proteins including membrane channels and intracellular transporters (Bronner, 2001). The pivotal role of these proteins is reflected by the great number of channelopathies caused by their dysfunction, such as many forms of neoplasia, epilepsy, neuropathic pain and neurodegeneration (Dolphin, 2016). Radiofrequency radiation has been reported to decrease pan‐Ca^2+^ channel expression in mouse hippocampus and hypothalamus, increase cytosolic [Ca^2+^] in stem‐cell derived neuronal cells and decrease Ca^2+^ binding proteins expression (Kim et al., 2018, 2019; Maskey et al., 2013; Titushkin et al., 2009). On the other hand, ELF‐EMFs have been linked to an increase of [Ca^2+^] in various cells and tissues, amongst which mouse hippocampus and rat PC12 cells (Morabito et al., 2010; Sun et al., 2016; Yin et al., 2016), enhance the expression of voltage‐gated calcium channels (VGCCs), and modulate their electrical properties (Duan et al., 2014; Lisi et al., 2006; Marchionni et al., 2006; Sun et al., 2016). However, results reported in literature are often conflicting and other studies report no effect on Ca^2+^ current or perturbation of Ca^2+^ homeostasis (Groot et al., 2014; Marchionni et al., 2006; Platano et al., 2007).

Additionally, the mechanism through which EMFs impact Ca^2+^ dynamics has yet to be unraveled. The numerous evidence reporting a change in the expression and transport dynamics of VGCCs suggests the cellular membrane as the preferential target for EMF interaction (Adey, 1993). Furthermore, many studies suggest the direct involvement of specific VGCCs, such as L, P/Q, N and T subtypes (Buckner et al., 2015; Lisi et al., 2006; Marchionni et al., 2006; Sun et al., 2016; Titushkin et al., 2009). However, the large number of discordant results cannot be overlooked (Groot et al., 2014, 2016; Platano et al., 2007), with several studies suggesting the alteration in Ca^2+^ homeostasis is independent of VGCC dynamics (Luo et al., 2014; Morabito et al., 2010).

HEK 293 cells are broadly used as a platform for the heterologous expression of diverse subtypes of Ca^2+^ channels, such as T‐type and L‐type, in the study of EMF exposure (Cui et al., 2014; Hristov et al., 2018) and they are particularly interesting due to their numerous neuronal characteristics such as sensitivity to the neurotransmitter acetylcholine, bradykinin and neurotensin (Vetter & Lewis, 2010). However, little is known about the endogenous response of HEK 293 cells to EMFs, as their native Ca^2+^ dynamics are still a matter of debate (Berjukow et al., 1996; Bugaj et al., 2005; Varghese et al., 2006).

Here, we aim to determine the specific mechanistic pathway involved in the alteration of internal Ca^2+^ homeostasis in HEK 293 cells, under exposure to RF‐EMFs and SMFs.

## 2 MATERIALS AND METHODS

### 2.1 Cell Culture

The human embryonic kidney cell line HEK 293 (Sigma‐Aldrich, UK) was cultured in high glucose Dulbecco's modified Eagle's medium (DMEM) (Thermo Fisher Scientific, UK), supplemented with 10% heat inactivated fetal bovine serum (Thermo Fisher Scientific, UK), 1% Penicillin/Streptomycin (Thermo Fisher Scientific, UK) and 1% L‐Glutamine (Sigma Aldrich, UK) stock solutions. On the day prior to each experiment cells (confluence 60–70%) were divided and seeded in 96‐well plates (Thermo Fisher Scientific, UK) on a volume of 100 µl/well. For some experiments, cells were directly seeded in Standard Extracellular Physiological Saline (120 mM NaCl, 4 mM KCl, 2mM CaCl_2_, 2 mM MgCl_2_, 10 mM HEPES, 10 mM glucose). Each well was checked for full confluence before the experiments.

### 2.2 Determination of intracellular [Ca^2+^]

**FIGURE 1:**
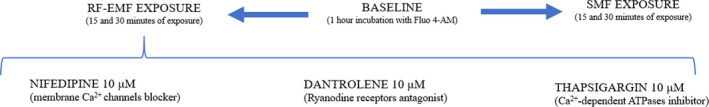
Flow chart of the experimental protocol. The impact on intracellular [Ca^2+^] of oscillating (RF‐EMFs) and static (SMFs) fields was initially assessed. To elucidate the origin of the detected increase, the RF‐EMFs experiments were then repeated by using different compounds. These were: 10 μM Nifedipine as Ca^2+^ channels blocker; 10 μM Thapsigargin as blocker of Ca^2+^‐dependent ATPases (SERCAs) inhibitor; 10 μM Dantrolene as antagonist of Ryanodine receptors of (RyRs)

Intracellular [Ca^2+^] changes were assessed using Fluo‐4 AM (Abcam, UK). Cells were seeded in two 96‐wells microplates (Thermo Fisher Scientific, UK) on the day prior to the experiment to obtain full confluence on experiment day. On experiment day, culture media was removed, and each plate washed with Standard Extracellular Physiological Saline (120 mM NaCl, 4 mM KCl, 2mM CaCl_2_, 2 mM MgCl_2_, 10 mM HEPES, 10 mM glucose) to remove any trace of dead cells or culture media. Both plates were then incubated for 1 h (38°C, 5% CO_2_) in Standard Extracellular Physiological Saline containing 1 μM Fluo‐4AM (Thermo Fisher Scientific, UK), 50 μl/well. After incubation time, plates were washed again and resuspended in 50 μl of Standard Extracellular Physiological Saline. A first measurement of intracellular [Ca^2+^] was acquired using a Clariostar Plate reader (BGM Labtech, Germany), pre‐adjusted for gain and focus level, and used as baseline. Plates then underwent exposure protocols (details in the below section). Fluorescence intensity reflecting intracellular [Ca^2+^] was measured at T_1_ (15 min) and T_2_ (30 min) (Figure 1). The baseline was subtracted to the measurement taken at each timepoint and the relative increases of the sham and the exposed groups compared.

To assess the origin of the detected increase, some experiments were performed in Ca^2+^‐free solution (120 mM NaCl, 4 mM KCl, 2 mM MgCl_2_, 10 mM HEPES, 10 mM glucose). To evaluate minimum responses, 10 µM of EGTA (Sigma‐Aldrich, UK) was also added to dye solution in some experiments. To interfere with intracellular and extracellular Ca^2+^ dynamics various compounds were also used and added to the dye incubation solution (Figure 1): as Ca^2+^ channel blocker,10 μM Nifedipine (Sigma‐Aldrich, UK); to block SERCAs on the ER, 10 μM Thapsigargin (Santa Cruz Biotechnology, USA); to block RyRs, 10 μM Dantrolene (Sigma‐Aldrich, UK). Data were recorded as individual well scans.

### 2.3 EMF exposure conditions

**FIGURE 2:**
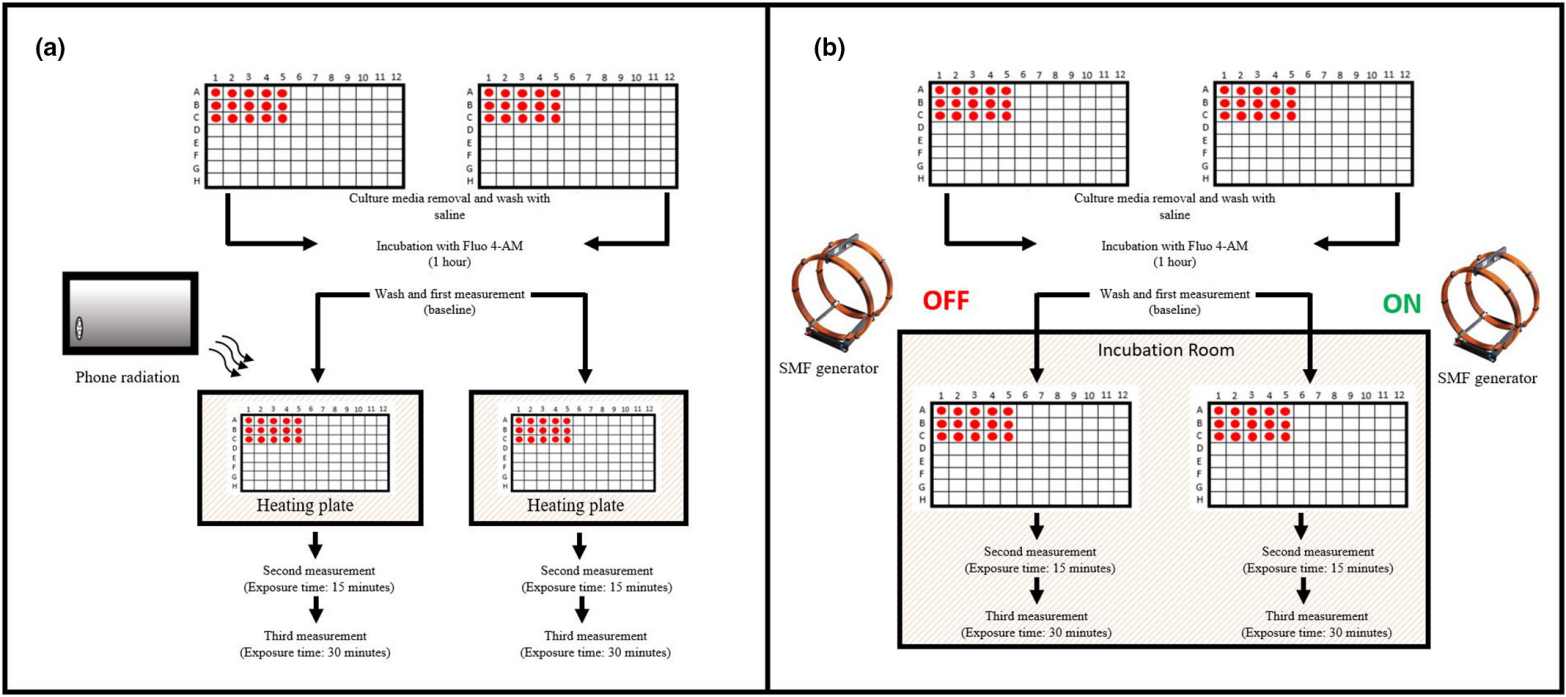
Experimental arrangement of RF‐EMF and SMF experiments: plates were incubated for 1 h with Fluo‐4AM Ca^2+^ dye. A first measurement was used as baseline. For RF‐EMF exposure (a), a cell phone was used as source of radiation. Both plates were then placed on a heating plate at 38°C. The cell phone's antenna was directly placed on the charged wells (red dots in the figure) for maximum radiation. Sham group was subjected to the same condition of the exposed group, but for the presence of the phone. For SMFs (b), we used a custom generator composed of two parallel Helmholtz coils powered by a DC supply. Both plates were incubated at a monitored temperature of 38°C. Sham group was subjected to the same condition of the exposed group, but the generator was turned off. In both cases, plates were exposed for 30 min and fluorescence intensity, reflecting intracellular [Ca^2+^], measured after 15 and 30 min

The sham group was submitted to the same conditions as the EMF group, but without EMF irradiation. In the RF‐EMFs experiments, a mobile telephone (Nord, OnePlus) was used as a source of radiation (Figure 2a). The mobile telephone was operated in test mode and was powered through a stabilized power supply, so that the antenna power supply as well as the field intensity were constant. In this diagnostic configuration the cell phone produced a constant field having a measured intensity of 0.4 mT. The maximum radiation was achieved in a 50 × 50 mm square corresponding to the location of the mobile telephone antenna. Plates were exposed for a total of 30 min and intracellular [Ca^2+^] evaluated every 15 min. For maximum radiation, the EMF source was placed directly on the top of plate's lid (Figure 2a). The Specific Absorption Rate (SAR) as stated by producing company (OnePlus) was 1.27 W/Kg. Both sham and exposed plates were placed throughout the whole experiment on a heating plate producing a measured average temperature of 38°C. A thermal probe was placed in a test microplate well, loaded with experimental solution and used to monitor the temperature over exposure to phone cell activity and SMF generator. Temperature was monitored over 30 min before each experiment and no major fluctuations were recorded as a consequence of exposure conditions. A thermal probe was placed in a test microplate well, loaded with experimental solution and used to monitor the temperature over exposure to phone cell activity and SMF generator. Temperature was monitored over 30 min before each experiment and no major fluctuations were recorded as a consequence of exposure conditions. In SMF experiments the plates were placed in a SMF generator consisting of two Helmholtz Coils (Figure 2b) powered by a direct current (DC) generator and used to produce a constant SMF of 1 mT (measured before and after each experiment). Differences smaller than ±1%, in respective to the center, in a cylindrical volume of 60 mm in diameter × 60 mm in length, minimum. The SMF generator was placed in an incubation room (38°C) for the duration of the experiments. The sham plate was subjected to the same conditions of the exposure groups, but the SMF generator was off.

### 2.4 Statistical Analysis

Statistical analysis was performed using Graph Pad Prism^®^ software version 6 for Windows (*p* < 0.05). Error bars represent standard error of the mean (SEM). T‐tests were used to determine whether a difference is seen between the means of two unrelated groups of data. The “*n*” value reported refer to microplate wells. For each condition 3 independent experiments were carried out.

All experimental procedures were approved by and adhered to the guidelines of ethical committee of University of Surrey, Guildford, UK.

## 3 RESULTS

### 3.1 EMFs triggers intracellular Ca^2+^ release

To compare the impact of the electric and the magnetic component of the exposure, the effect of different frequency fields was assessed. Specifically, the exposures consisted of oscillating radiofrequency fields (RF‐EMFs) and static magnetic field (SMFs), constant in time and direction, and thus, with a 0 Hz frequency. For the first we choose to use a mobile phone, closely resembling the exposure deriving from smartphones and wireless devices usage. For the second, a static magnetic field generator consisting of two Helmholtz coils connected to a DC power supply. As we were interested in the rapid effects of EMFs, we focused our investigations on early timepoints (15 and 30 min).

A rise in dye fluorescence, reflecting an increase in intracellular [Ca^2+^], was observed as result of both types of exposures. In the RF‐EMF exposed cohort, this was significantly different at T_1_ (15 min) and T_2_ (30 min) (*p*‐value < 0.0001) when compared to sham. Additionally, in this group when compared between T_1_ and T_2_, the increase in intracellular Ca^2+^ was significantly increased (*p*‐value 0.0002) in T_2_ when compared to T_1_ suggesting a time dependent effect on intracellular increases albeit acute.

**FIGURE 3:**
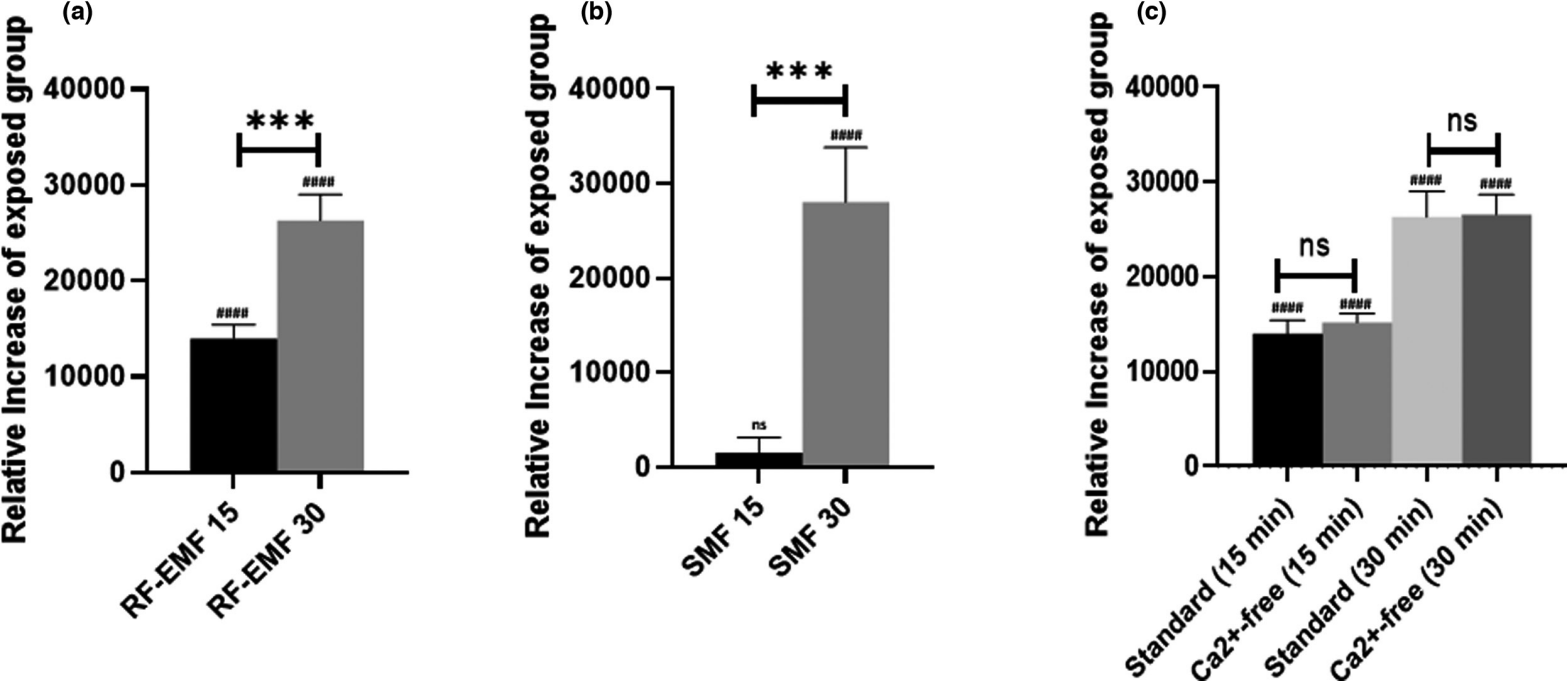
SMF and RF‐EMFs increase intracellular [Ca^2+^] in HEK 293 cells. Data are displayed as relative increase of exposed group when compared to sham. (*) represent inter‐conditions significance while (#) display significance when compared to sham. (a) RF‐EMFs increase basal [Ca^2+^] The difference with sham group is significant at both T_1_ and T_2_. (b) SMFs lead to a similar increase as already found with RF‐EMFs. However, the increase is delayed when compared to what observed with RF‐EMFs, and only significant at T_2_. (c) Comparison between RF‐EMF elicited increase in standard and Ca^2+^‐free conditions. The extracellular presence of Ca^2+^ makes no difference on the detected [Ca^2+^]_i_, suggesting an intracellular origin for the elicited increase. *N* = 45 microplate wells from 3 independent experiments. *p*‐value < 0.001

The SMFs, on the other hand, showed no difference between sham and exposed groups at T_1_, but at T_2_, the exposure showed a significant increase (*p*‐value < 0.0001) when compared to the sham (Figure 3b). Similar to the RF‐EMF group, when compared between T_1_ and T_2_, the intracellular Ca^2+^ was significantly increased in T_2_ (*p*‐value 0.0009) when compared to T_1_, also suggesting a time dependent effect on intracellular Ca^2+^ increase. The temperature generated by phone cell activity and SMF generator were monitored with a thermal probe over 30 min before experiments. Since in both cases they were always lower than the incubating temperature of the plates (37.5°C), the thermal effects were considered to be not significant. No significant difference in temperature was detected when the SMF generator was switched on or turned off.

As, contrary to SMFs, acute exposure to RF‐EMFs is not commonly reported to increase cytosolic [Ca^2+^], we decided to focus our investigation on this type of fields. In order to characterize the origin of the detected increase, the RF‐EMF experiments were repeated in Ca^2+^‐free solutions, aiming to evaluate the involvement of intracellular Ca^2+^ stores. We observed no significant difference between Ca^2+^‐free and standard physiological solution at both T_1_ and T_2_. This suggested an intracellular origin for the RF‐EMF‐elicited increase in Ca^2+^.

### 3.2 Confirmation of the involvement of membrane Ca^2+^ channels

Further experiments were undertaken to clarify the exact mechanism for the observed change in intracellular Ca^2+^. In order to investigate the involvement of membrane Ca^2+^channels, cells were administered with 10 μM of the Ca^2+^ channel blocker Nifedipine. All the drugs were directly added to the dye solution so that their impact was evaluated both when compared to baseline and to the field exposures.

**FIGURE 4:**
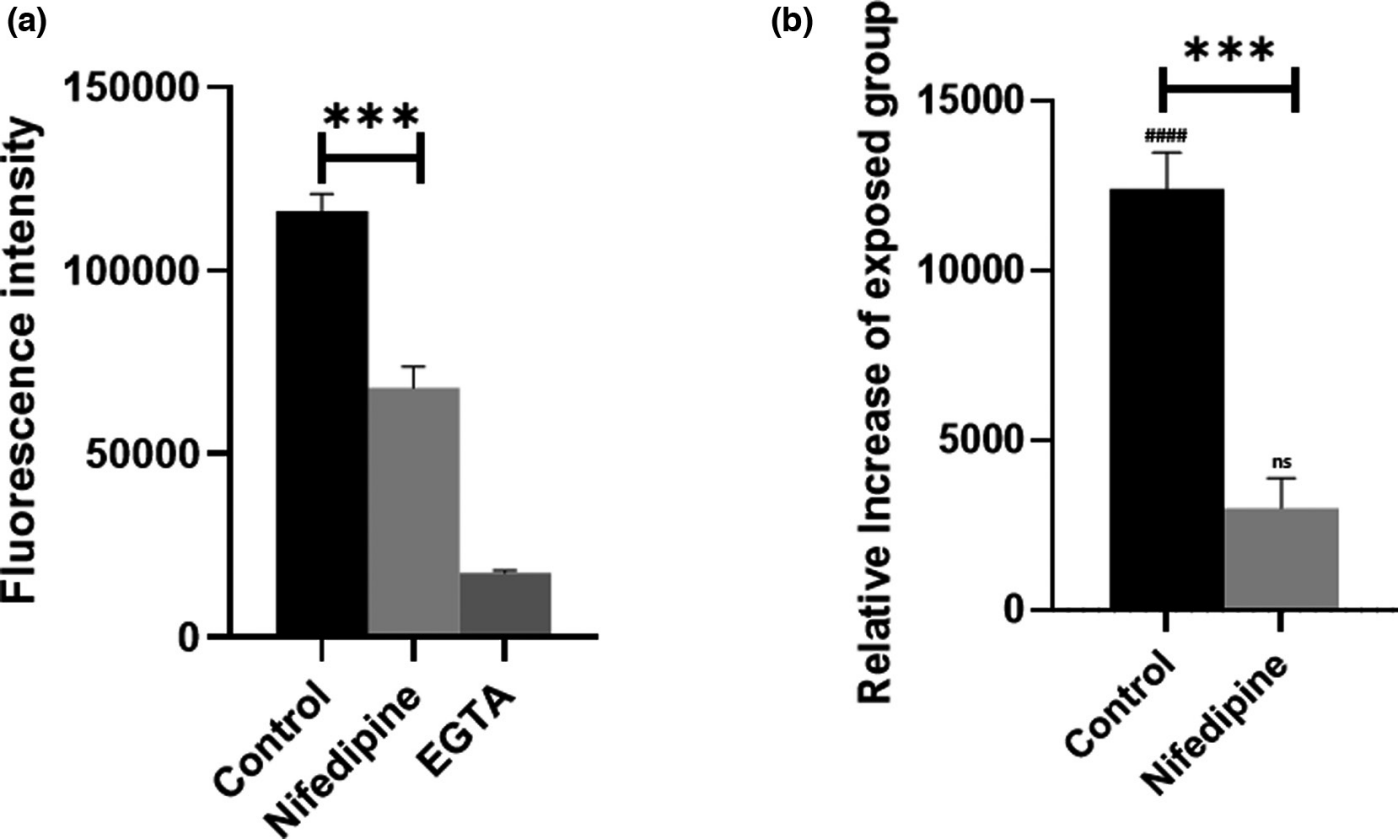
Nifedipine administration confirms the presence of Nifedipine‐sensitive channels on the membrane of HEK 293 cells, as their involvement in the increase in intracellular Ca^2+^ elicited by RF‐EMFs. Data in (b) are displayed as relative increase of exposed group when compared to sham. (*) represent inter‐condition significance while (#) display significance when compared to sham. (a) The intracellular [Ca^2+^] is lowered by the addition of 10 μM Nifedipine. Response to Ca^2+^ chelator EGTA was observed to be minimal. (b) Administration of 10 μM Nifedipine is sufficient to null any difference between sham and exposed group at T_2_ exposure. *N* = 45 microplate wells from 3 independent experiments. *p*‐value < 0.001

Nifedipine treatment significantly reduced intracellular [Ca^2+^] (*p*‐value 0.0002), confirming the presence of nifedipine‐sensitive channels on the membrane of HEK 293 cells (Figure 4a). Moreover, it strongly reduced the increase of the exposed group when compared to control (*p*‐value < 0.0001), suggesting the involvement of these channels in the modulation of the increase triggered by EMFs exposure (Figure 4b). The bigger reduction of fluorescence intensity achieved in the EGTA‐treated group in respect to Nifedipine‐treated one proves that a significant fraction of intracellular Ca^2+^ is likely to originate from intracellular reservoirs, or it is translocated through nifedipine‐insensitive channels.

### 3.3 Blockage of ER replenishment impairs RF‐EMF effects

**FIGURE 5:**
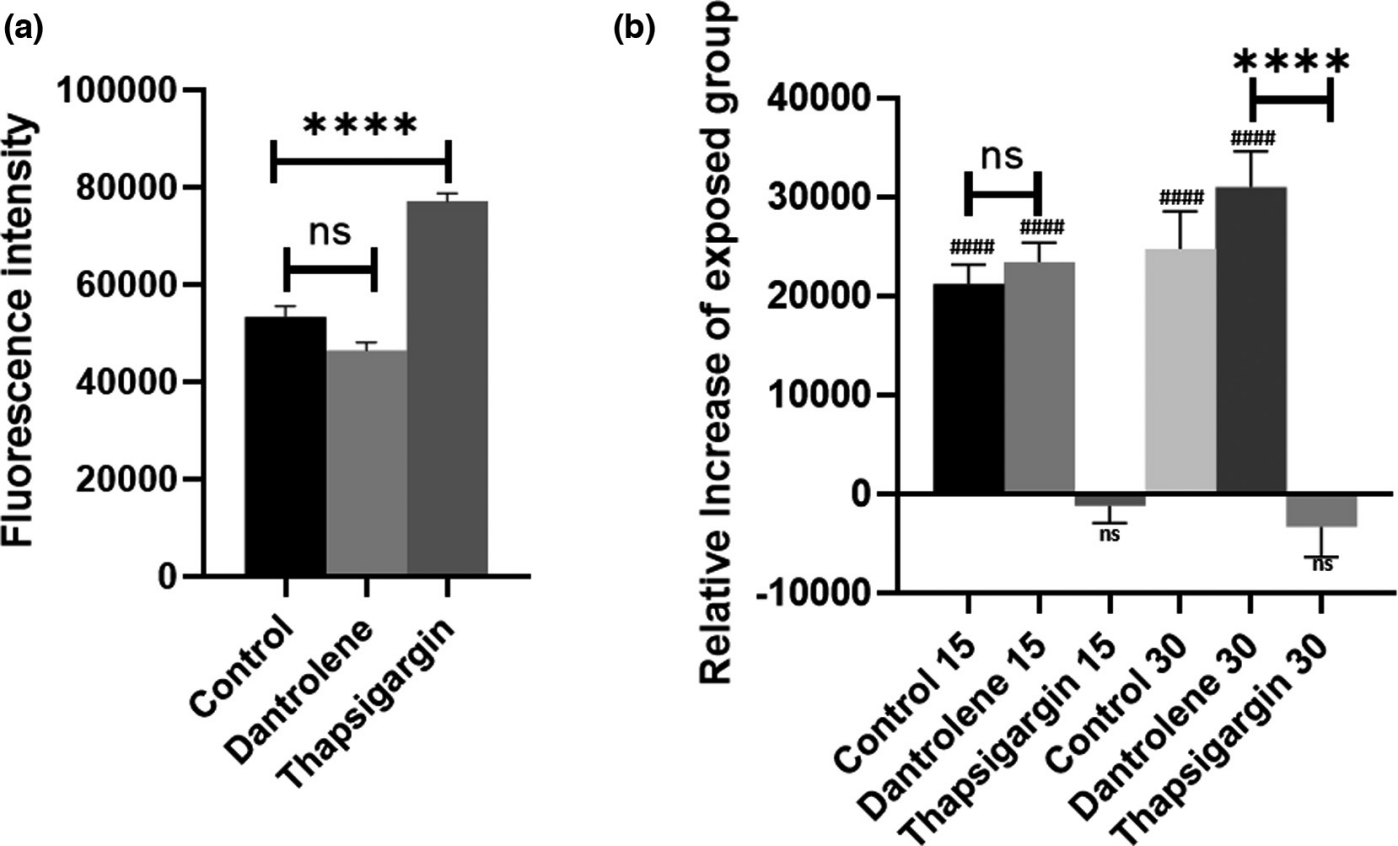
Blockage of ER replenishment impairs RF‐EMFs response. Data in (b) are displayed as relative increase of exposed group when compared to sham. (a) Baseline after incubation with 10 μM Dantrolene and 10 μM Thapsigargin. The block of SERCAs operated by Thapsigargin raise intracellular Ca^2+^, impairing ER replenishment. The effect of dantrolene is not significant in line with the poor functional expression of RyRs in HEK 293 cells. (b) Administration of Thapsigargin is sufficient to completely null any difference between sham and exposed group at both T_1_ and T_2_ Dantrolene treated group displays no significant difference when compared to control. *N* = 45 microplate wells from 3 independent experiments. *p*‐value < 0.001

The impact of Nifedipine, mainly thought to target cell membrane Ca^2+^ channels, was apparently in contrast with the intracellular origin suggested by the Ca^2+^‐free experiments described above. However, Nifedipine was also shown to interfere with the dynamics of intracellular Ca^2+^ stores (Curtis & Scholfield, 2001; Rosales & Brown, 1992), mainly represented, in non‐excitable cells, by the endoplasmic reticulum (ER). In order to elucidate the role of intracellular Ca^2+^ stores, we decided to target the ER‐cytoplasm Ca^2+^ exchanges at different levels. In the ER, Ca^2+^ release is mainly mediated by the activity of Ryanodine Receptors (RyRs). Hence, we assessed the impact of Dantrolene (10 μM), a commonly used antagonist of RyRs. Dantrolene treated group displayed no difference when compared to the control, both in the sham (Figure 5a) and exposed group (Figure 5b).

On the other hand, cytoplasmic Ca^2+^ withdrawal is mainly mediated by sarco/endoplasmic reticulum Ca^2+^‐ATPases (SERCAs). These transporters are responsible for the replenishment of ER stores by consequence of the release of Ca^2+^ in the cytoplasm. To inhibit the activity of SERCAs, cells were treated with 10 μM Thapsigargin, a commonly used SERCA blocker. An initial intracellular Ca^2+^ increase was detected (*p*‐value < 0.0001), in line with the blockage of ER replenishment as with the activation of non‐selective Ca^2+^‐permeable cation channels triggered by depletion of intracellular stores. Strikingly, in this group the increase at both T_1_ and T_2_ exposure was minimal, and no significant difference was observed between the two timepoints. Moreover, the relative increase was significantly different (*p*‐value < 0.0001) to that of control and dantrolene‐treated group, both at T_1_ and T_2_ (Figure 5b). This highlights the direct involvement of an internal Ca^2+^ release, deriving from the ER, in the intracellular Ca^2+^ increase elicited by EMFs.

All together these results suggest a cell membrane‐independent mechanism, involving the ER intracellular Ca^2+^ release in the modulations of Ca^2+^ homeostasis perturbations elicited by EMFs.

## 4 DISCUSSION

Our investigation on EMFs effect on intracellular [Ca^2+^] in HEK 293 cells revealed an important link between exposure to both static and oscillating magnetic fields and augmented presence of cytoplasmic Ca^2+^. This outcome agrees with the abundant evidence found in literature about this correlation (Duan et al., 2014; Luo et al., 2014; Morabito et al., 2010; Prina‐Mello et al., 2006). It is, however, in conflict with the results of other studies, showing no effect on basal [Ca^2+^] as consequence of exposure to RF‐EMFs (O'Connor et al., 2010). This discrepancy could be due to the extreme variability of effects elicited by electromagnetic radiation, that is thought to be dependent on the frequency and the intensity of the field, the exposure time and the model studied (Bertagna et al., 2021). As a consequence, the results of this study have to be related to the particular protocol used and cell line studied here. Nonetheless, the comparison between the two different exposure platforms used here (RF‐EMFs and SMFs) supports the finding indicating oscillating EMFs, characterized by a continuous electromagnetic wave, to be more biologically active than SMFs, in virtue of their greater perturbation of electromagnetic homeostasis of the cell (Panagopoulos et al., 2015).

The relative concentrations of Ca^2+^ in different subcellular compartments are finely regulated by different types of transporters, amongst which ion channels are thought to play a primary role (Carafoli & Crompton, 1978). In line with this, an involvement of these proteins in the modulation of EMF biological effects is well established. Indeed, the particular sensitivity of ion channels, as their role in controlling the different ionic concentration underlying the majority of cellular signaling pathways, makes them a perfect target for EMF effects (Funk et al., 2009). This is particularly evident in excitable tissues expressing voltage‐gated ion channels (VGCs) as the central nervous system, where an electromagnetic wave, mainly in virtue of its electrical field component, can easily displace the charges on the protein voltage sensor, modifying the gating dynamics and the conductance of the channels themselves (Bertagna et al., 2021; Mathie et al., 2003). Both RF‐EMFs and SMFs have been shown to affect VGC activity in many ways, including their expression, presence in the membrane, gating and inactivation dynamics (Kim et al., 2018, 2019; Marchionni et al., 2006; Shen et al., 2007; Sun et al., 2016).

Despite their pivotal contribution, the dynamics of intracellular Ca^2+^ exchanges are not exclusively determined by cell membrane channel activity and thus may be affected by EMFs at different levels, partially or totally independent by cell membrane dynamics. Indeed, as fluctuations in intracellular Ca^2+^ results are the most commonly reported effect of EMF exposure (Bertagna et al., 2021), many studies state these perturbations to be independent by cell membrane dynamics (Luo et al., 2014; Morabito et al., 2010). Here, we observed an increase in intracellular [Ca^2+^] in Ca^2+^‐free conditions similar to the one reported in standard physiological conditions. This suggested an intracellular origin for the increase in Ca^2+^. Importantly, a disruption in internal Ca^2+^ homeostasis is linked to the activation of apoptotic pathways and numerous other pathways involved in cell survival (Orrenius et al., 2003). Notably, changes in apoptotic and autophagic pathways, are commonly reported as a result of EMF exposure (Kim et al., 2018), albeit this modulation could be a consequence of Ca^2+^ homeostasis disturbance.

Here, we focused on the role of the endoplasmic reticulum (ER) that, along with mitochondria, is primarily involved in the control of cytoplasmic [Ca^2+^]. Dantrolene treatment was proven to have no effect on the EMF‐elicited Ca^2+^ increase, displaying no difference when compared to the control group that received the same type of exposure. However, this could be due to the poor functional expression of endogenous RyRs in HEK 293 cells, as previously shown by Western Blotting analysis (Tong et al., 1999). The functional presence of RyRs in HEK 293 cells is matter of debate, as different studies report conflicting results (Querfurth et al., 1998; Tong et al., 1999). Nonetheless, our results seem to support a limited involvement of these transporters in the intracellular Ca^2+^ ER‐cytoplasm efflux of HEK 293 cells, that could be conversely controlled by other proteins, as inositol 1,4,5‐trisphosphate receptor (IP_3_R) (Aoyama et al., 2004). It will be therefore important, in future investigations, to assess the role of these proteins in EMF‐elicited Ca^2+^ modulation.

We were able to null EMF response by impairing the replenishment of the ER through the administration of 10 μM Thapsigargin, a specific blocker SERCAs. Despite the presence of Ca^2+^ in the external solution, the intracellular Ca^2+^ was mostly abolished, suggesting a direct involvement of the ER release in the detected Ca^2+^ raise. Moreover, no difference was observed between sham and exposed group, indicating a direct involvement of the ER internal stores in the EMFs modulation. These results are in line with both the involvement of ER in the intracellular Ca^2+^ increase detected and with the involvement of a membrane independent mechanism, modulating EMF effects, as already observed as consequence of exposure to extremely low‐frequency fields in entorhinal cortex neurons (Luo et al., 2014).

Noteworthily, the ER stress response is one of the numerous signaling cascades modulated by Ca^2+^. This pathway is initiated within ER and was found to be critical for cell survival. ER stress is accompanied by alterations in Ca^2+^ homeostasis and altered level of reactive oxygen species (ROS). Interestingly, altered oxidative stress has been linked to both ER abnormal function and different fields exposure (Morabito et al., 2010; Pooam et al., 2020), as to the pathogenesis of multiple human disorders, as cancer and neurodegenerative diseases, that seem to be also modulated by EMFs (Chio & Tuveson, 2017; Van Raamsdonk et al., 2017).

It is important to stress the early timepoints on which we focused this study. The rapid effects observed are in line with a broad literature reporting basal Ca^2+^ increase as result of acute (<24 h) exposure to different frequencies and intensities of EMFs, but different, and sometimes opposite, effects are reported for chronic exposure. This controversy could be maybe resolved by considering the time‐dependent cellular adaptation to perturbances of Ca^2+^ homeostasis, that could involve both fast and slow regulations, as different (positive and negative) feedback loops.

A consensus on the specific contribution of Ca^2+^ internal stores in EMF‐elicited alteration of Ca^2+^ homeostasis is still missing, as diverse mechanisms involving different cellular compartments could have a different impact depending on the tissue. Nonetheless, this research contributes to the understanding of the cellular effect elicited by EMF on internal Ca^2+^ dynamics of HEK 293 cells and sheds a light on the key involvement of the ER in this modulation.

## CONFLICT OF INTEREST

The authors declare no competing interests.

## AUTHOR CONTRIBUTION

F.B., K.J., R.L., J.M., and S.R.P.S. designed the research. F.B., K.J., and R.L. analyzed the data. F.B. performed experiments. F.B., K.J., and R.L. wrote the paper. All authors approved the final manuscript.

## References

[phy215189-bib-0001] Adey, W. R. (1993). Biological effects of electromagnetic fields. Journal of Cellular Biochemistry, 51, 410–416. 10.1002/jcb.2400510405 8388394

[phy215189-bib-0002] Aoyama, M. , Yamada, A. , Wang, J. , Ohya, S. , Furuzono, S. , Goto, T. , Hotta, S. , Ito, Y. , Matsubara, T. , Shimokata, K. , Chen, S. R. W. , Imaizumi, Y. , & Nakayama, S. (2004). Requirement of ryanodine receptors for pacemaker Ca2+ activity in ICC and HEK293 cells. Journal of Cell Science, 117, 2813–2825. 10.1242/jcs.01136 15169838

[phy215189-bib-0003] Baan, R. , Grosse, Y. , Lauby‐Secretan, B. , El Ghissassi, F. , Bouvard, V. , Benbrahim‐Tallaa, L. , Guha, N. , Islami, F. , Galichet, L. , & Straif, K. (2011). Carcinogenicity of radiofrequency electromagnetic fields. The Lancet Oncology, 12, 624–626. 10.1016/S1470-2045(11)70147-4 21845765

[phy215189-bib-0004] Balassa, T. , Varró, P. , Elek, S. , Drozdovszky, O. , Szemerszky, R. , Világi, I. , & Bárdos, G. (2013). Changes in synaptic efficacy in rat brain slices following extremely low‐frequency magnetic field exposure at embryonic and early postnatal age. International Journal of Developmental Neuroscience, 31, 724–730. 10.1016/j.ijdevneu.2013.08.004 24012627

[phy215189-bib-0005] Berjukow, S. , Döring, F. , Froschmayr, M. , Grabner, M. , Glossmann, H. , & Hering, S. (1996). Endogenous calcium channels in human embryonic kidney (HEK293) cells. British Journal of Pharmacology, 118, 748–754. 10.1111/j.1476-5381.1996.tb15463.x 8762103 PMC1909701

[phy215189-bib-0006] Bertagna, F. , Lewis, R. , Silva, S. R. P. , McFadden, J. , & Jeevaratnam, K. (2021). Effects of electromagnetic fields on neuronal ion channels: A systematic review. Annals of the New York Academy of Sciences, 1499(1), 82–103. 10.1111/nyas.14597 33945157

[phy215189-bib-0007] Bronner, F. (2001). Extracellular and intracellular regulation of calcium homeostasis. TheScientificWorldJournal, 1, 919–925. 10.1100/tsw.2001.489 PMC6084056 12805727

[phy215189-bib-0008] Buckner, C. A. , Buckner, A. L. , Koren, S. A. , Persinger, M. A. , & Lafrenie, R. M. (2015). Inhibition of cancer cell growth by exposure to a specific time‐varying electromagnetic field involves T‐type calcium channels. PLoS One, 10, e0124136. 10.1371/journal.pone.0124136 25875081 PMC4397079

[phy215189-bib-0009] Bugaj, V. , Alexeenko, V. , Zubov, A. , Glushankova, L. , Nikolaev, A. , Wang, Z. , Kaznacheyeva, E. , Bezprozvanny, I. , & Mozhayeva, G. N. (2005). Functional properties of endogenous receptor‐and store‐operated calcium influx channels in HEK293 cells. Journal of Biological Chemistry, 280, 16790–16797. 10.1074/jbc.M500192200 15741171

[phy215189-bib-0010] Carafoli, E. , & Crompton, M. (1978). The regulation of intracellular calcium. Current topics in membranes and transport, Vol. 10 (pp. 151–216). Elsevier.

[phy215189-bib-0011] Chio, I. I. C. , & Tuveson, D. A. (2017). ROS in cancer: the burning question. Trends in Molecular Medicine, 23, 411–429. 10.1016/j.molmed.2017.03.004 28427863 PMC5462452

[phy215189-bib-0012] Choi, J. , Min, K. , Jeon, S. , Kim, N. , Pack, J.‐K. , Song, K. (2020). Continuous exposure to 1.7 GHz LTE electromagnetic fields increases intracellular reactive oxygen species to decrease human cell proliferation and induce senescence. Scientific Reports, 10, 1–15. 32514068 10.1038/s41598-020-65732-4 PMC7280220

[phy215189-bib-0013] Cui, Y. , Liu, X. , Yang, T. , Mei, Y.‐A. , & Hu, C. (2014). Exposure to extremely low‐frequency electromagnetic fields inhibits T‐type calcium channels via AA/LTE4 signaling pathway. Cell Calcium, 55, 48–58. 10.1016/j.ceca.2013.11.002 24360572

[phy215189-bib-0014] Curtis, T. M. , & Scholfield, C. N. (2001). Nifedipine blocks Ca2+ store refilling through a pathway not involving L‐type Ca2+ channels in rabbit arteriolar smooth muscle. The Journal of Physiology, 532, 609–623. 11313433 10.1111/j.1469-7793.2001.0609e.x PMC2278590

[phy215189-bib-0015] Czyz, J. , Guan, K. , Zeng, Q. , Nikolova, T. , Meister, A. , Schönborn, F. , Schuderer, J. , Kuster, N. , Wobus A.M . (2004). High frequency electromagnetic fields (GSM signals) affect gene expression levels in tumor suppressor p53‐deficient embryonic stem cells. Bioelectromagnetics: Journal of the Bioelectromagnetics Society. The Society for Physical Regulation in Biology and Medicine, the European Bioelectromagnetics Association, 25, 296–307. 10.1002/bem.10199 15114639

[phy215189-bib-0016] de Groot, M. W. , Kock, M. D. , & Westerink, R. H. (2014). Assessment of the neurotoxic potential of exposure to 50 Hz extremely low frequency electromagnetic fields (ELF‐EMF) in naive and chemically stressed PC12 cells. Neurotoxicology, 44, 358–364. 25111744 10.1016/j.neuro.2014.07.009

[phy215189-bib-0017] de Groot, M. W. , van Kleef, R. G. , de Groot, A. , Westerink, R.H. (2016). In vitro developmental neurotoxicity following chronic exposure to 50 Hz extremely low‐frequency electromagnetic fields in primary rat cortical cultures. Toxicological Sciences, 149, 433–440. 26572663 10.1093/toxsci/kfv242

[phy215189-bib-0018] Dolphin, A. C. (2016). Voltage‐gated calcium channels and their auxiliary subunits: Physiology and pathophysiology and pharmacology. The Journal of Physiology, 594, 5369–5390. 10.1113/JP272262 27273705 PMC5043047

[phy215189-bib-0019] Duan, Y. , Wang, Z. , Zhang, H. , He, Y. ; Fan, R. ; Cheng, Y. , Sun, G. , Sun, X. (2014). Extremely low frequency electromagnetic field exposure causes cognitive impairment associated with alteration of the glutamate level, MAPK pathway activation and decreased CREB phosphorylation in mice hippocampus: Reversal by procyanidins extracted from the lotus seedpod. Food & Function, 5, 2289–2297. 25066354 10.1039/c4fo00250d

[phy215189-bib-0020] Ebashi, S. , & Endo, M. (1968). Calcium and muscle contraction. Progress in Biophysics and Molecular Biology, 18, 123–183. 10.1016/0079-6107(68)90023-0 4894870

[phy215189-bib-0021] Funk, R. H. , Monsees, T. , & Özkucur, N. (2009). Electromagnetic effects–From cell biology to medicine. Progress in Histochemistry and Cytochemistry, 43, 177–264. 10.1016/j.proghi.2008.07.001 19167986

[phy215189-bib-0022] Grassi, C. , D’Ascenzo, M. , Torsello, A. , Martinotti, G. , Wolf, F. , Cittadini, A. , & Azzena, G. B. (2004). Effects of 50 Hz electromagnetic fields on voltage‐gated Ca2+ channels and their role in modulation of neuroendocrine cell proliferation and death. Cell Calcium, 35, 307–315. 10.1016/j.ceca.2003.09.001 15036948

[phy215189-bib-0023] Haghani, M. , Shabani, M. , & Moazzami, K. (2013). Maternal mobile phone exposure adversely affects the electrophysiological properties of Purkinje neurons in rat offspring. Neuroscience, 250, 588–598. 10.1016/j.neuroscience.2013.07.049 23906636

[phy215189-bib-0024] Hallett, M. (2000). Transcranial magnetic stimulation and the human brain. Nature, 406, 147–150. 10.1038/35018000 10910346

[phy215189-bib-0025] Hardingham, G. E. , Chawla, S. , Johnson, C. M. , & Bading, H. (1997). Distinct functions of nuclear and cytoplasmic calcium in the control of gene expression. Nature, 385, 260–265. 10.1038/385260a0 9000075

[phy215189-bib-0026] He, Y.‐L. , Liu, D.‐D. , Fang, Y.‐J. , Zhan, X.‐Q. , Yao, J.‐J. , & Mei, Y.‐A. (2013). Exposure to extremely low‐frequency electromagnetic fields modulates Na+ currents in rat cerebellar granule cells through increase of AA/PGE2 and EP receptor‐mediated cAMP/PKA pathway. PLoS One, 8, e54376. 10.1371/journal.pone.0054376 23349866 PMC3551899

[phy215189-bib-0027] Hristov, K. , Mangalanathan, U. , Casciola, M. , Pakhomova, O. N. , & Pakhomov, A. G. (2018). Expression of voltage‐gated calcium channels augments cell susceptibility to membrane disruption by nanosecond pulsed electric field. Biochimica Et Biophysica Acta (BBA)‐Biomembranes, 1860, 2175–2183. 10.1016/j.bbamem.2018.08.017 30409513

[phy215189-bib-0100] IARC Working Group on the Evaluation of Carcinogenic Risks to Humans, World Health Organization, and International Agency for Research on Cancer (2002) Non‐ionizing Radiation: Static and extremely low‐frequency (ELF) electric and magnetic fields. PMC5098132 12071196

[phy215189-bib-0029] Jimenez, H. , Blackman, C. , Lesser, G. , Debinski, W. , Chan, M. , Sharma, S. , Watabe, K. , Lo, H.‐W. , Thomas, A. , Godwin, D. (2018). Use of non‐ionizing electromagnetic fields for the treatment of cancer. Frontiers in Bioscience, 23, 284–297. 10.2741/4591 28930547

[phy215189-bib-0030] Kim, J. H. , Huh, Y. H. , & Kim, H. R. (2019). Trafficking of synaptic vesicles is changed at the hypothalamus by exposure to an 835 MHz radiofrequency electromagnetic field. General Physiology and Biophysics, 38, 379–388. 10.4149/gpb_2019020 31411574

[phy215189-bib-0031] Kim, J. H. , Sohn, U. D. , Kim, H.‐G. , Kim, H.R. (2018). Exposure to 835 MHz RF‐EMF decreases the expression of calcium channels, inhibits apoptosis, but induces autophagy in the mouse hippocampus. The Korean Journal of Physiology & Pharmacology, 22, 277. 29719450 10.4196/kjpp.2018.22.3.277 PMC5928341

[phy215189-bib-0032] Lacy‐hulbert, A. , Metcalfe, J. C. , & Hesketh, R. (1998). Biological responses to electromagnetic fields 1. The FASEB Journal, 12, 395–420. 9535213 10.1096/fasebj.12.6.395

[phy215189-bib-0033] Lisi, A. , Ledda, M. , Rosola, E. , Pozzi, D. , Emilia, E.D. , Giuliani, L. , Foletti, A. , Modesti, A. , Morris, S.J. , Grimaldi, S. (2006). Extremely low frequency electromagnetic field exposure promotes differentiation of pituitary corticotrope‐derived AtT20 D16V cells. Bioelectromagnetics: Journal of the Bioelectromagnetics Society. The Society for Physical Regulation in Biology and Medicine, the European Bioelectromagnetics Association, 27, 641–651. 10.1002/bem.20255 16838272

[phy215189-bib-0034] London, S. J. , Thomas, D. C. , Bowman, J. D. , Sobel, E. , Cheng, T.‐C. , & Peters, J. M. (1991). Exposure to residential electric and magnetic fields and risk of childhood leukemia. American Journal of Epidemiology, 134, 923–937. 10.1093/oxfordjournals.aje.a116176 1843457

[phy215189-bib-0035] Luo, F.‐L. , Yang, N. , He, C. , Li, H.‐L. , Li, C. , Chen, F. , Xiong, J.‐X. , Hu, Z.‐A. , & Zhang, J. (2014). Exposure to extremely low frequency electromagnetic fields alters the calcium dynamics of cultured entorhinal cortex neurons. Environmental Research, 135, 236–246. 10.1016/j.envres.2014.09.023 25462671

[phy215189-bib-0036] Marchionni, I. , Paffi, A. , Pellegrino, M. , Liberti, M. , Apollonio, F. , Abeti, R. , Fontana, F. , d'Inzeo, G. , Mazzanti, M. (2006). Comparison between low‐level 50 Hz and 900 MHz electromagnetic stimulation on single channel ionic currents and on firing frequency in dorsal root ganglion isolated neurons. Biochimica Et Biophysica Acta (BBA)‐Biomembranes, 1758, 597–605. 16713990 10.1016/j.bbamem.2006.03.014

[phy215189-bib-0037] Markov, M. S. (2007). Expanding use of pulsed electromagnetic field therapies. Electromagnetic Biology and Medicine, 26, 257–274. 10.1080/15368370701580806 17886012

[phy215189-bib-0038] Martiny, K. , Lunde, M. , & Bech, P. (2010). Transcranial low voltage pulsed electromagnetic fields in patients with treatment‐resistant depression. Biological Psychiatry, 68, 163–169. 10.1016/j.biopsych.2010.02.017 20385376

[phy215189-bib-0039] Maskey, D. , Lee, J.‐K. , Kim, H. R. , & Kim, H.‐G. (2013). Neuroprotective effect of ginseng against alteration of calcium binding proteins immunoreactivity in the mice hippocampus after radiofrequency exposure. BioMed Research International, 2013, 1–12. 10.1155/2013/812641 PMC3773416 24069603

[phy215189-bib-0040] Mathie, A. , Kennard, L. E. , & Veale, E. L. (2003). Neuronal ion channels and their sensitivity to extremely low frequency weak electric field effects. Radiation Protection Dosimetry, 106, 311–315. 10.1093/oxfordjournals.rpd.a006365 14690272

[phy215189-bib-0041] Morabito, C. , Guarnieri, S. , Fanò, G. , & Mariggiò, M. A. (2010). Effects of acute and chronic low frequency electromagnetic field exposure on PC12 cells during neuronal differentiation. Cellular Physiology and Biochemistry, 26, 947–958. 10.1159/000324003 21220925

[phy215189-bib-0042] Neher, E. , & Sakaba, T. (2008). Multiple roles of calcium ions in the regulation of neurotransmitter release. Neuron, 59, 861–872. 10.1016/j.neuron.2008.08.019 18817727

[phy215189-bib-0043] Nicotera, P. , & Orrenius, S. (1998). The role of calcium in apoptosis. Cell Calcium, 23, 173–180. 10.1016/S0143-4160(98)90116-6 9601613

[phy215189-bib-0044] O'Connor, R. P. , Madison, S. D. , Leveque, P. , Roderick, H.L. , Bootman, M.D. (2010). Exposure to GSM RF fields does not affect calcium homeostasis in human endothelial cells, rat pheocromocytoma cells or rat hippocampal neurons. PLoS One, 5, e11828. 20676401 10.1371/journal.pone.0011828 PMC2910734

[phy215189-bib-0045] Odaci, E. , Bas, O. , & Kaplan, S. (2008). Effects of prenatal exposure to a 900 MHz electromagnetic field on the dentate gyrus of rats: a stereological and histopathological study. Brain Research, 1238, 224–229. 18761003 10.1016/j.brainres.2008.08.013

[phy215189-bib-0046] Organization, W.H . (2007). Electromagnetic fields and public health: Exposure to extremely low frequency fields‐fact sheet N 322. Retrieved April 9, 2010.

[phy215189-bib-0047] Orrenius, S. , Zhivotovsky, B. , & Nicotera, P. (2003). Regulation of cell death: the calcium–apoptosis link. Nature Reviews Molecular Cell Biology, 4, 552–565. 10.1038/nrm1150 12838338

[phy215189-bib-0048] Panagopoulos, D. J. , Johansson, O. , & Carlo, G. L. (2015). Real versus simulated mobile phone exposures in experimental studies. BioMed Research International, 2015, 1–8. 10.1155/2015/607053 PMC4539441 26346766

[phy215189-bib-0049] Platano, D. , Mesirca, P. , Paffi, A. , Pellegrino, M. , Liberti, M. , Apollonio, F. , Bersani, F. , Aicardi, G. (2007). Acute exposure to low‐level CW and GSM‐modulated 900 MHz radiofrequency does not affect Ba2+ currents through voltage‐gated calcium channels in rat cortical neurons. Bioelectromagnetics: Journal of the Bioelectromagnetics Society. The Society for Physical Regulation in Biology and Medicine, the European Bioelectromagnetics Association, 28, 599–607. 10.1002/bem.20345 17620299

[phy215189-bib-0050] Pooam, M. , Jourdan, N. , El Esawi, M. , Sherrard, R. M. , & Ahmad, M. (2020). HEK293 cell response to static magnetic fields via the radical pair mechanism may explain therapeutic effects of pulsed electromagnetic fields. PLoS One, 15, e0243038. 10.1371/journal.pone.0243038 33270696 PMC7714230

[phy215189-bib-0051] Prina‐Mello, A. , Farrell, E. , Prendergast, P. , Campbell, V. , Coey, J. (2006). Influence of strong static magnetic fields on primary cortical neurons. Bioelectromagnetics: Journal of the Bioelectromagnetics Society. The Society for Physical Regulation in Biology and Medicine, the European Bioelectromagnetics Association, 27, 35–42. 10.1002/bem.20173 16283651

[phy215189-bib-0052] Querfurth, H. W. , Haughey, N. J. , Greenway, S. C. , Yacono, P. W. , Golan, D. E. , & Geiger, J. D. (1998). Expression of ryanodine receptors in human embryonic kidney (HEK293) cells. Biochemical Journal, 334, 79–86. 10.1042/bj3340079 PMC1219664 9693105

[phy215189-bib-0053] Reddy, A. S. , Ali, G. S. , Celesnik, H. , & Day, I. S. (2011). Coping with stresses: Roles of calcium‐and calcium/calmodulin‐regulated gene expression. The Plant Cell, 23, 2010–2032. 10.1105/tpc.111.084988 21642548 PMC3159525

[phy215189-bib-0054] Rosales, C. , & Brown, E. (1992). Calcium channel blockers nifedipine and diltiazem inhibit Ca2+ release from intracellular stores in neutrophils. Journal of Biological Chemistry, 267, 1443–1448. 10.1016/S0021-9258(18)45965-0 1730694

[phy215189-bib-0055] Savitz, D. A. , Wachtel, H. , Barnes, F. A. , John, E. M. , & Tvrdik, J. G. (1988). Case‐control study of childhood cancer and exposure to 60‐Hz magnetic fields. American Journal of Epidemiology, 128, 21–38. 10.1093/oxfordjournals.aje.a114943 3164167

[phy215189-bib-0056] Shen, J.‐F. , Chao, Y.‐L. , & Du, L. (2007). Effects of static magnetic fields on the voltage‐gated potassium channel currents in trigeminal root ganglion neurons. Neuroscience Letters, 415, 164–168. 10.1016/j.neulet.2007.01.015 17289262

[phy215189-bib-0057] Sun, Z.‐C. , Ge, J.‐L. , Guo, B. , Guo, J. , Hao, M. , Wu, Y.‐C. , Lin, Y.‐A. , La, T. , Yao, P.‐T. , Mei, Y.‐A. , Feng, Y. , & Xue, L. (2016). Extremely low frequency electromagnetic fields facilitate vesicle endocytosis by increasing presynaptic calcium channel expression at a central synapse. Scientific Reports, 6, 1–11. 10.1038/srep21774 26887777 PMC4757866

[phy215189-bib-0058] Tian, D. , Jacobo, S. M. , Billing, D. , Rozkalne, A. , Gage, S.D. , Anagnostou, T. , Pavenstädt, H. , Hsu, H.‐H., Schlondorff, J. , Ramos, A. (2010). Antagonistic regulation of actin dynamics and cell motility by TRPC5 and TRPC6 channels. Science Signaling, 3, ra77. 10.1126/scisignal.2001200 20978238 PMC3071756

[phy215189-bib-0059] Titushkin, I. , Rao, V. , Pickard, W. , Moros, E. , Shafirstein, G. , Cho, M. (2009). Altered calcium dynamics mediates P19‐derived neuron‐like cell responses to millimeter‐wave radiation. Radiation Research, 172, 725–736. 10.1667/RR1760.1 19929419

[phy215189-bib-0060] Tong, J. , Du, G. G. , Chen, S. R. W. , & Maclennan, D. H. (1999). HEK‐293 cells possess a carbachol‐and thapsigargin‐sensitive intracellular Ca2+ store that is responsive to stop‐flow medium changes and insensitive to caffeine and ryanodine. Biochemical Journal, 343, 39–44. 10.1042/bj3430039 PMC1220521 10493909

[phy215189-bib-0061] Tsien, R. (1983). Calcium channels in excitable cell membranes. Annual Review of Physiology, 45, 341–358. 10.1146/annurev.ph.45.030183.002013 6303205

[phy215189-bib-0062] Van Raamsdonk, J. M. , Vega, I. E. , & Brundin, P. (2017). Oxidative stress in neurodegenerative disease: causation or association? Oncotarget, 8, 10777. 10.18632/oncotarget.14650 28099897 PMC5355220

[phy215189-bib-0063] Varghese, A. , TenBroek, E. M. , Coles, J. Jr , & Sigg, D. (2006). Endogenous channels in HEK cells and potential roles in HCN ionic current measurements. Progress in Biophysics and Molecular Biology, 90, 26–37. 10.1016/j.pbiomolbio.2005.05.002 15979128

[phy215189-bib-0064] Vetter, I. , & Lewis, R. J. (2010). Characterization of endogenous calcium responses in neuronal cell lines. Biochemical Pharmacology, 79, 908–920. 10.1016/j.bcp.2009.10.020 19883631

[phy215189-bib-0065] Wyszkowska, J. , Jankowska, M. , & Gas, P. (2019). Electromagnetic fields and neurodegenerative diseases. Przegląd Elektrotechniczny, 1, 129–133. 10.15199/48.2019.01.33

[phy215189-bib-0066] Yin, C. , Luo, X. , Duan, Y. , Duan, W. , Zhang, H. , He, Y. , Sun, G. , & Sun, X. (2016). Neuroprotective effects of lotus seedpod procyanidins on extremely low frequency electromagnetic field‐induced neurotoxicity in primary cultured hippocampal neurons. Biomedicine & Pharmacotherapy, 82, 628–639. 10.1016/j.biopha.2016.05.032 27470406

